# High-resolution NMR characterization of low abundance oligomers of amyloid-β without purification

**DOI:** 10.1038/srep11811

**Published:** 2015-07-03

**Authors:** Samuel A. Kotler, Jeffrey R. Brender, Subramanian Vivekanandan, Yuta Suzuki, Kazutoshi Yamamoto, Martine Monette, Janarthanan Krishnamoorthy, Patrick Walsh, Meagan Cauble, Mark M. Banaszak Holl, E. Neil. G. Marsh, Ayyalusamy Ramamoorthy

**Affiliations:** 1 Biophysics, University of Michigan-Ann Arbor, Ann Arbor, Michigan 48109, U.S.A; 2 Department of Chemistry, University of Michigan-Ann Arbor, Ann Arbor, Michigan 48109, U.S.A; 3 Michigan Nanotechnology Institute for Medicine and Biological Sciences, University of Michigan-Ann Arbor, Ann Arbor, Michigan 48109, U.S.A; 4 Bruker BioSpin Ltd., Bruker Corporation, 555 E Steeles Ave, Milton, ON, Canada

## Abstract

Alzheimer’s disease is characterized by the misfolding and self-assembly of the amyloidogenic protein amyloid-β (Aβ). The aggregation of Aβ leads to diverse oligomeric states, each of which may be potential targets for intervention. Obtaining insight into Aβ oligomers at the atomic level has been a major challenge to most techniques. Here, we use magic angle spinning recoupling ^1^H-^1^H NMR experiments to overcome many of these limitations. Using ^1^H-^1^H dipolar couplings as a NMR spectral filter to remove both high and low molecular weight species, we provide atomic-level characterization of a non-fibrillar aggregation product of the Aβ_1-40_ peptide using non-frozen samples without isotopic labeling. Importantly, this spectral filter allows the detection of the specific oligomer signal without a separate purification procedure. In comparison to other solid-state NMR techniques, the experiment is extraordinarily selective and sensitive. A resolved 2D spectra could be acquired of a small population of oligomers (6 micrograms, 7% of the total) amongst a much larger population of monomers and fibers (93% of the total). By coupling real-time ^1^H-^1^H NMR experiments with other biophysical measurements, we show that a stable, primarily disordered Aβ_1-40_ oligomer 5–15 nm in diameter can form and coexist in parallel with the well-known cross-β-sheet fibrils.

Alzheimer’s disease (AD) is a fatal neurological disorder affecting more than five million people in the United States today; a figure that is expected to increase three-fold by 2050 if therapeutics remain inadequate^1^. Although the exact cause of AD remains undetermined, many signs point to the involvement of the aggregation of the amyloid-β (Aβ) peptide at some stage^2^,^3^. The aggregation of Aβ leads to the formation of senile plaques found in patients with AD, the main constituent of which is the Aβ peptide in its fibrillar form^4^. However, attempts at pharmaceutical intervention aimed at targeting Aβ aggregation has been complicated by the myriad forms that aggregates of Aβ can adopt, many of which remain poorly characterized^3^,^5^,^6^.

Much effort has been undertaken in the way of understanding the structural details of the monomeric^7^,^8^,^9^ and fibrillar^10^,^11^,^12^,^13^ forms of Aβ both computationally and experimentally; however, there are only few existing structural models of the intermediates formed along the misfolding pathway of Aβ^14^,^15^,^16^,^17^. Unfortunately, these are the species currently believed to be most critical for pathogenesis in Alzheimer’s and other amyloid related neurodegenerative diseases^3^. Most of the models that do exist bear a close structural resemblance to the fiber end-product^15^,^18^,^19^,^20^, with few exceptions^21^. However, considerable evidence from lower-resolution techniques like CD suggests some (but not all) of the most toxic oligomers may have a considerably different structure that may be closer to the Aβ monomer than the Aβ fiber^22^,^23^,^24^,^25^.

The transient nature and high heterogeneity of amyloid oligomers present significant challenges for high-resolution structural studies. Oligomers of a specific conformation are difficult to isolate, which has thus far severely limited high-resolution characterization of Aβ. Moreover, oligomer structures of Aβ_1-40_ and Aβ_1-42_ have not been obtained by crystallography (only a low resolution structure of a specific Aβ oligomer obtained by powder X-ray diffraction and modeling exists)^26^, nor have they been obtained for oligomers of other amyloidogenic proteins, except for αB-crystallin^27^.

Previous studies have shown Aβ_1-40_’s ability to form unordered, globular aggregates with little to no secondary structure^22^,^24^,^25^,^28^. Not only are these oligomers a critical step in the aggregation of Aβ, but they also exhibit a high degree of cytotoxicity^22^,^24^,^25^. A time dependent ^19^F NMR study showed that such oligomers can persist even after prolonged incubation (>50 days) and the formation of amyloid fibrils^29^; however, none of these studies demonstrated structural details beyond low-resolution measurements. To characterize the disordered globular oligomers, we chose experimental conditions similar to these previous studies using low salt concentrations and neutral pH, yet here we also apply agitation during seeded fibrillization. This method of preparation results in the formation of an aggregated Aβ sample comprised mostly of fibrils with a small population of a predominantly disordered oligomeric species of Aβ_1-40_. Remarkably, this method of preparation yielded disordered oligomers reproducibly at almost 10% of the total peptide concentration without perturbative methods, such as chemical- or photo-crosslinking, freezing, amino acid substitution, or any other type of protein engineering to stabilize the oligomer.

Using magic angle spinning (MAS) NMR spectroscopy on these samples, we are able to resolve structural details otherwise unobservable by other biophysical measurements. We do so by bridging the gap in the limitations imposed on solution- and solid-state NMR methods; a molecular weight limit in solution NMR and problems with sensitivity in solid-state NMR. These limitations are overcome by taking advantage of both solution- and solid-state NMR techniques: performing solid-state NMR experiments on a liquid sample with “solid” characteristics. A similar approach was taken with a solution sample of Aβ by monitoring the formation and kinetics of large aggregates sedimenting out of solution^30^. However, this technique used ^13^C detection requiring large amounts of sample and expensive, isotopic labeling.

Here, we employ a RFDR (radio frequency driven dipolar recoupling)^31^ -based 2D ^1^H/^1^H chemical shift correlation experiment to overcome these limitations. High-resolution structural features of high molecular weight oligomers are difficult to characterize directly by solution NMR; however, the RFDR-based 2D ^1^H/^1^H experiment enables the specific detection of this oligomeric species over fibrillar and monomeric Aβ_1-40_ due to the line-narrowing afforded by slow MAS and the reintroduction of residual ^1^H-^1^H dipolar couplings by RFDR. Previously, the applicability of RFDR for the study of such “soft” solid systems was used to investigate the structural and motional characteristics of a resin-bound peptide^32^, a micelle-associated cytochrome b5^33^, and membrane-bound peptides^34^,^35^,^36^. Here, we demonstrate the utility of ^1^H/^1^H RFDR for the specific structural characterization of a minority population of a stable, disordered Aβ_1-40_ oligomer containing sparse secondary structure, at low abundance, directly from an unlabeled sample in coexistence with amyloid fibrils without further purification or filtration. To the best of our knowledge, this is the first instance in which ^1^H-^1^H dipolar couplings have been used for structural studies of an amyloid oligomer, which may provide a general method to study intermediate size oligomers of the type believed to play a crucial role in amyloid pathology^3^.

## Results and discussion

### Aβ_1-40_ can form a disordered oligomer in parallel with β-sheet fibrils

**Figure 1:**
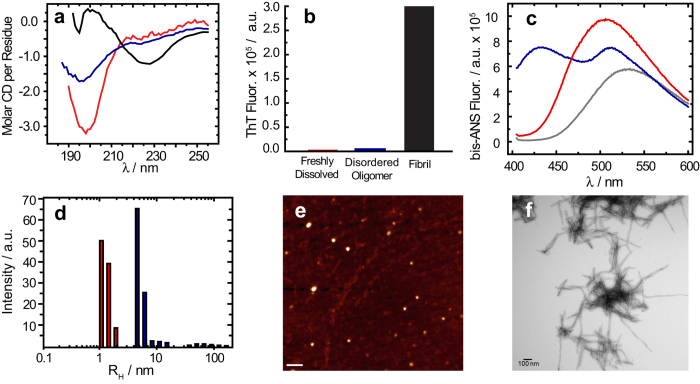
Biophysical characterization of Aβ_1-40_ disordered oligomers. (**a**) CD spectra, (**b**) ThT fluorescence, and (**c**) bis-ANS fluorescence of fibrillar (black), the spin-X-isolated oligomer (blue), and freshly dissolved (red) samples of Aβ_1-40_. In panel c, the emission spectrum of bis-ANS in solution is shown in grey. (**d**) Distributions of the hydrodynamic radii of the freshly dissolved Aβ_1-40_ (red) and the spin-X-isolated Aβ_1-40_ oligomer (blue) determined by DLS. Representative AFM and TEM images of the Aβ_1-40_ oligomers (**e**) and fibrils (**f**). Scale bars in both images are 100 nm. All experiments were performed in 10 mM sodium phosphate buffer, pH 7.4 at 25  °C.

Since intermediate size (<100 kDa) oligomers have been a prime target of both pharmaceutical and scientific research^3^, we first sought to find a condition that allowed the reproducible generation of oligomers of this type. Our previous study utilizing real-time observation of a single-site ^19^F-label at the Met-35 of Aβ_1-40_, found that Aβ_1-40_ oligomers are observed and stabilized well into the late stages of the aggregation process^29^. In agreement with Suzuki *et al.*^29^, we find the coexistence of oligomers and fibers in our samples; however, the oligomers formed under our shaking conditions are not of the β-sheet type. A CD spectrum (Fig. 1a, solid) shows an intensity minimum at 225 nm indicating the bulk sample is predominantly β-sheet. Using size-cutoff filtration with a Spin-X 0.22 μm filter, we could isolate the oligomers and any residual monomers from the fibrils of Aβ_1-40._ A noticeably different CD spectrum is apparent in the filtrate, which shows a strong minimum near 198 nm indicative of a predominantly disordered structure (Fig. 1a, blue) very similar to that of early aggregation states of Aβ (Fig. 1a, red). We further confirmed the lack of β-sheet content of the filtered Aβ_1-40_ oligomers using the amyloid-specific dye, ThT (Fig. 1b). Similar to the freshly dissolved Aβ_1-40_ sample, the oligomers isolated by spin-X filtration display essentially no fluorescence in its ThT emission profile, indicating a β-sheet conformation is not present (Fig. 1b). Conversely, an intense ThT signal is present in the isolated fibril fraction. Measuring the concentration of the spin-X filtrate by the bicinchoninic acid (BCA) protein concentration assay (Thermo Scientific) revealed 17.0 ± 6.0 μM or only 7.3 + 2.6% of the total Aβ_1-40_ concentration (231 μM) was not fibrillar. Together, the ThT, CD, and BCA results confirm the sample primarily consists of amyloid fibrils with a minority population of a largely disordered, relatively low MW species.

Since the CD spectra of the spin-X filtrate resemble CD spectra of monomeric Aβ, it is possible that the filtered sample consists of residual monomeric Aβ_1-40_. We first tested this possibility with the molecular probe 4,4′-Dianilino-1,1′-Binaphthyl-5,5′-Disulfonic Acid (bis-ANS). The spectral properties and the quantum yield of bis-ANS are highly sensitive to polarity, thus upon binding to hydrophobic surfaces bis-ANS becomes appreciably more fluorescent. A unique feature of bis-ANS is its ability to selectively identify different aggregation states of Aβ_1-40_ through distinct emission spectra (Fig. 1c)^22^,^37^,^38^. The emission spectra of bis-ANS after isolation and separation of the fibrils from disordered oligomers is shown in Fig. 1c, along with the bis-ANS signal from a freshly dissolved Aβ_1-40_ sample prepared without incubation or seeding. The emission spectrum of bis-ANS in the spin-X-isolated oligomers (Fig. 1c, blue) is significantly more blue-shifted than that of the emission spectrum observed from the fibril fraction (Supplementary Fig. 1). Also in Fig. 1c, the bis-ANS fluorescence observed in the presence of the freshly dissolved Aβ_1-40_ sample (red) is only slightly blue-shifted from that of the spectrum of bis-ANS alone (black), indicating limited binding of bis-ANS to monomeric Aβ_1-40_ and a significant difference in the hydrophobic exposure compared to the freshly dissolved Aβ_1-40_ or the Aβ_1-40_ fibrils.

In addition to the bis-ANS measurements, dynamic light scattering (DLS) studies revealed distinct size distributions of these disordered Aβ oligomers when compared to the freshly dissolved Aβ_1-40_ sample (Fig. 1d and Supplementary Fig. 2). DLS measured a R_H_ of ~5.1 nm with a polydispersity of 32.2% for the oligomers isolated by spin-X filtration, whereas the R_H_ of the freshly dissolved Aβ_1-40_ sample was ~1.4 nm with a polydispersity of 19.0%. To complement our optical spectroscopy data, representative electron micrographs and AFM images of the amyloid preparations showed oligomeric and fibrillar forms present in the aggregated sample of Aβ_1-40_ (Fig. 1e,f). All AFM images showed the disordered Aβ oligomers exhibit an amorphous, spherical morphology (Fig. 1e). The oligomers were found present among a dense fibrillar network whose bundled, twisted features were apparent in all electron micrographs as observed in Fig. 1f. Taken together, all biophysical measurements point to the existence of a disordered and soluble Aβ_1-40_ oligomer coexisting as a minority population amongst Aβ_1-40_ fibrils. This finding is very intriguing as it indicates Aβ_1-40_ is simultaneously undergoing β-sheet and non-β-sheet aggregation pathways. For this reason, we aimed to find distinct structural features that contributed to the formation of such an Aβ oligomer.

### The RFDR-based 2D ^1^H/^1^H chemical shift correlation provides site-specific information on a minority population of disordered Aβ_1-40_ oligomers in a fiber-containing sample

**Figure 2:**
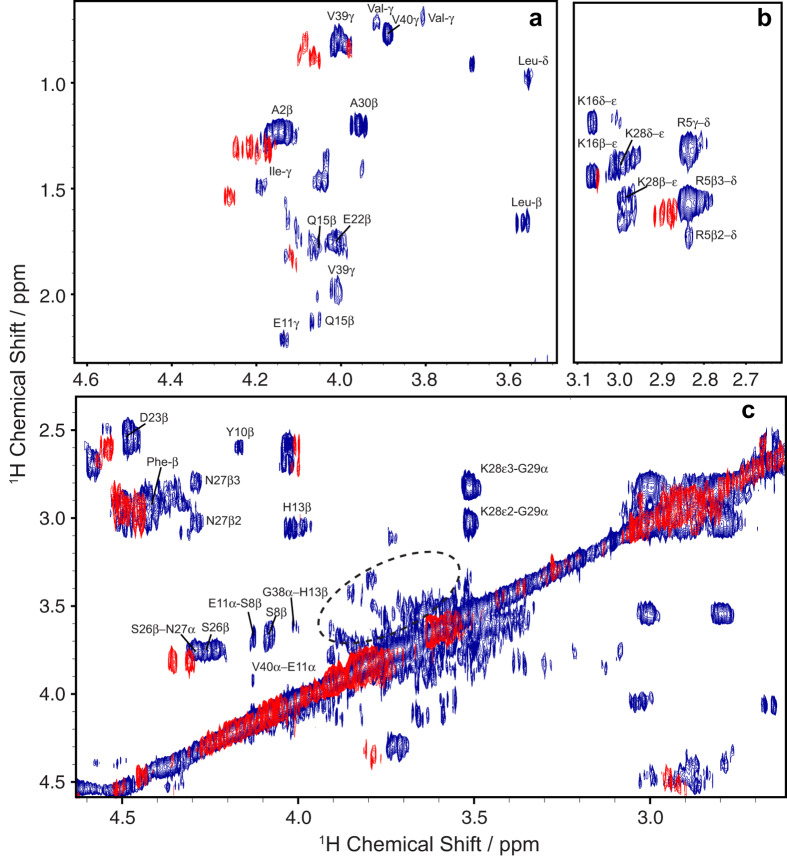
RFDR-based 2D ^1^H/^1^H chemical shift correlation spectra of freshly dissolved (red) and aggregated (blue) forms of Aβ_1-40_. (**a**) Side-chain to Hα, (**b**) side-chain, and (**c**) Hβ-Hα and Hα -Hα regions of the overlaid 2D spectra were recorded under 2.7 kHz MAS. The dotted circle highlights the Ser and Gly fingerprints of the aggregated Aβ_1-40_ sample. Peak assignments are given for the mixed Aβ_1-40_ sample. The spectra were acquired with a 50 ms mixing time at 600 MHz in 100% D_2_O, 10 mM sodium phosphate buffer, pH 7.4, and 37 °C. Total Aβ_1-40_ concentrations for both samples were 462 μM; the estimated oligomer concentration in the aggregated sample is 35 ± 12 μM. The acquisition time was 4 days.

To obtain an atomic-level picture of these disordered oligomers, we use a combination of solution- and solid-state NMR experiments. RFDR is different from the NOESY experiment in that the incorporation of rotor-synchronized π-pulses during the mixing period reintroduces coherent homonuclear dipole-dipole interactions (Supplementary Fig. 3). We first performed RFDR-based 2D ^1^H/^1^H experiments on two types of samples: the unfiltered, aggregated Aβ_1-40_ sample used in the CD experiments and a control sample of freshly dissolved Aβ_1-40_ without fibers made primarily of monomeric and low MW species. An overlay of the two 2D RFDR spectra is shown in Fig. 2. Only a few cross-peaks are observed in the 2D ^1^H/^1^H spectrum of the freshly dissolved Aβ_1-40_ sample (red in Fig. 2), probably due to the rapid tumbling of monomeric and/or low molecular weight Aβ_1-40_. The few cross-peaks that do appear in the spectrum of freshly dissolved Aβ_1-40_ likely arise from early aggregates, and the presence of peaks appearing near 0 ppm in the 1D ^1^H spectrum suggests that this is indeed the case (Supplementary Fig. 4). Such peaks (near 0 ppm) are commonly observed in the spectra of amyloidogenic peptides, and have been shown to occur due to the presence of an oligomeric species in which aliphatic protons are solvent protected and thus shifted to the high field region of the 1D ^1^H spectrum^39^,^40^. However, with the exception of these few cross-peaks, the RFDR-based 2D ^1^H/^1^H spectra of unaggregated Aβ_1-40_ is very sparse, consistent with weak (to negligible) ^1^H-^1^H dipolar couplings in low MW samples. The RFDR-based 2D ^1^H/^1^H experiment therefore acts as an efficient spectral filter for intermediate sized oligomers; low MW exhibit few cross-peaks because of weak (to negligible) ^1^H-^1^H dipolar couplings while the linewidth from very high MW species like amyloid fibers is too large (due to very strong dipolar couplings) to generate a detectable signal under the slow MAS speeds used in this study.

In contrast to the sparse spectra obtained from unaggregated Aβ_1-40,_ the RFDR-based 2D ^1^H/^1^H spectra of the aggregated sample showed multiple cross-peaks consistent with the presence of the disordered oligomer suggested by the CD, fluorescence, and AFM experiments (Fig.1). The dominant feature of the RFDR-based 2D ^1^H/^1^H spectra is a very strong upfield shift of Hα resonances compared to both the expected random coil values and the unaggregated, primarily monomeric sample. Upfield shifts typically derive from two primary sources: the formation of helical secondary structure and the shielding of the residue from solvent. Unfortunately, the spectra in Fig.2 are not sufficient to distinguish between these two sources.

However, this spectrum was taken under rather extreme conditions of low concentration (~35 μM) and high heterogeneity (the oligomers only constitute 7–10% of the entire sample) to test the limits of the technique to samples not traditionally considered amenable for NMR. To see if additional structural details could be resolved in a more conformationally pure sample, we performed the RFDR-based 2D ^1^H/^1^H experiment on purified oligomers by removing the fibers through Spin-X filtration using 10% D_2_O instead of 100% D_2_O to resolve the amide resonances. We first tested the influence of the MAS rate by increasing it up to 15 kHz (Supplementary Fig. 5). The fact that the resolution of 1D ^1^H spectra does not improve with the increasing MAS rate suggests that oligomers are not sedimenting out of solution at the speeds used in the experiment, although the selective sedimentation of fibers may play a role in enhancing the resolution of the mixed fiber/oligomer sample^30^.

**Figure 3:**
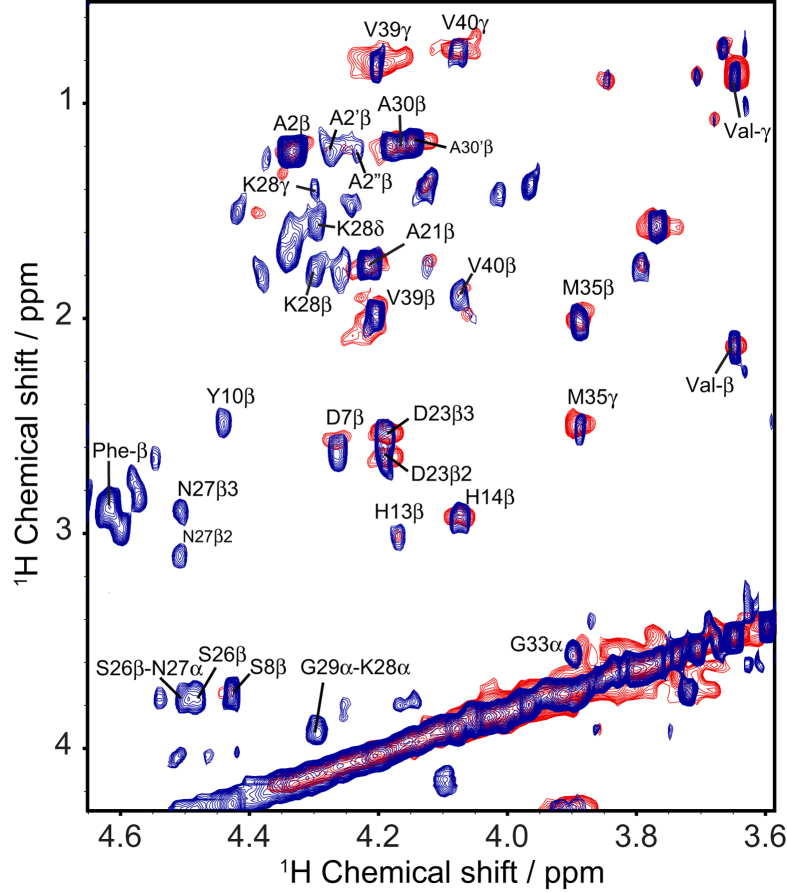
MAS spectra of the filtered disordered Aβ_1-40_ oligomer. An overlay of the assigned Hα regions from RFDR-based 2D ^1^H/^1^H (blue) and 2D ^1^H/^1^H TOCSY (red) spectra acquired at 298 K with 10 kHz MAS. The filtered oligomer sample was lyophilized and re-hydrated to double its initial concentration, making for a total Aβ_1-40_ of ~35 μM. The RFDR and TOCSY based 2D ^1^H/^1^H spectra were acquired with mixing times of 50 and 70 ms, respectively, at 600 MHz in 100% D_2_O, 10 mM sodium phosphate buffer, pH 7.4. RFDR spectra were acquired at 25 °C under 10 kHz MAS. Assignments are given for the RFDR-based 2D ^1^H/^1^H spectra. The non-uniform sampling based data acquisition time was 4 hours.

Under 10 kHz MAS, we were able to obtain a well-resolved RFDR-based 2D ^1^H/^1^H spectrum of the pure oligomer sample (Fig. 3). A majority of peaks assigned in the RFDR-based 2D ^1^H/^1^H spectrum of the unfiltered sample (Fig.2) are identified in the spectrum of the filtered oligomer sample as well (Fig. 3), suggesting peaks appearing in both samples arise from conformationally similar species. Under these conditions, it was possible to perform a partial assignment of the resonances using TOCSY experiments under MAS (Fig. 3, red spectrum). The lack of complete connectivity hampered the complete assignment of peaks in RFDR-based 2D ^1^H/^1^H spectra. Accordingly, the assignments of a partially folded structure of Aβ_1-40_ guided unambiguous assignment of 2D ^1^H/^1^H NMR spectra^14^. While the lack of connectivity is unavoidable given these constraints, the results suggest a spectrum that, while unusual for either a well-folded or unstructured protein, is consistent with results obtained from other biophysical experiments shown in Fig. 1. At the atomic level, the RFDR-based 2D ^1^H/^1^H spectrum contains inter-residue cross-peaks between the aliphatic and alpha protons of K28-G29, S26-N27, H13-G38, and S8-E11. These inter-residue contacts are observed for both 20 and 50 ms RFDR mixing times (Supplementary Fig. 6), indicating the interaction of these residues are prominent features for the oligomer’s structure. Moreover, the Ser and Gly fingerprints are observed only for the disordered oligomer species (circled cross-peaks in Fig. 2), suggesting the involvement of these residues in the oligomer formation and stabilization. More generally, though there are very strong up-field shifts for the resonances that usually suggest helix formation, no medium range αN (*i*, *i + 3*/*i + 4*) connectivity was found indicative of an α- or 3_10_- helical conformation. Rather, the strong upfield shift appears to be the result of the oligomerization of peptide and the consequent shielding of the residues from solvent. Taken together, this finding suggests a dynamic and disordered structure with a high degree of turns and twists but without a well-defined secondary structure.

We do not rule out the possibility that highly mobile regions of the fibril may contribute in part to the RFDR spectrum recorded on the mixed fibril-oligomer sample. The serine and glycine fingerprints highlighted in Fig. 2 are not found in the RFDR spectra of the filtered oligomer nor are they found in the freshly dissolved samples of Aβ_1-40_. Nevertheless, a majority of the cross-peaks assigned in the RFDR spectra of the filtered oligomer are the same as those assigned in the unfiltered Aβ_1-40_ sample. Given that any highly mobile residue from the Aβ fibril would largely maintain the fibril’s correlation time, it is more than likely that cross-peaks observed in the RFDR spectrum of the unfiltered sample derive from the disordered Aβ_1-40_ oligomer as MAS experiments performed on the filtered oligomer (Fig. 3 and Supplemental Fig. 5). Isolation of the oligomer, although helpful in improving the resolution (compare Fig. 2 and Fig. 3), is not strictly necessary.

### The disordered Aβ_1-40_ oligomer is conformationally stable and grows in size

**Figure 4:**
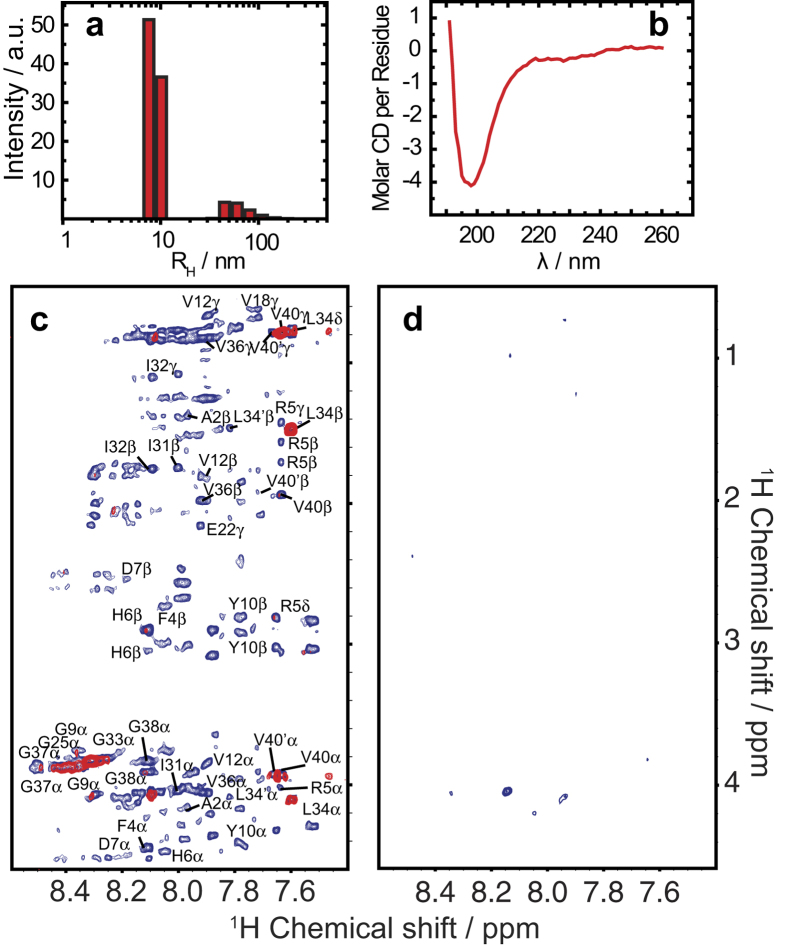
The disordered Aβ_1-40_ oligomer grows in size while maintaining its morphology. (**a**) DLS experiments at 19 days demonstrate that the disordered oligomer not only remains disordered, but also grows in size as well with a distribution of hydrodynamic radii at 8.6 nm and 65.3 nm and polydispersity of 14.9% and 37.8%, respectively. (**b**) The strong minimum at ~200 nm in the CD spectrum of the disordered oligomer after 19 days reveals that the oligomer does not progress to a fibrillar state. Two-dimensional spectra of the fingerprint region of (**c**) TOCSY (70 ms mixing) and (d) NOESY (250 ms mixing) of the disordered Aβ_1–40_ oligomers recorded at 4 days (blue) and 19 days (red). The filtered oligomer sample was lyophilized and re-hydrated to double its initial concentration, making for a total Aβ_1-40_ of ~35 μM. After 19 days (red), almost all peaks are broadened beyond detection in both TOCSY and NOESY spectra.

In light of our ability to purify the disordered Aβ oligomer, we were interested in whether the oligomers would aggregate into a fibrillar state. CD and DLS measurements confirm that the disordered nature of the oligomer is maintained while increasing in size over the course of 19 days (Fig. 4a,b). The CD spectrum of the spin-X-isolated oligomer at 19 days (Fig. 4b) shows a minimum intensity ca. 198 nm, indicative of random coil structure, and the DLS experiments indicate an increase in hydrodynamic radius of the oligomer from ~5.1 nm to ~8.6 nm (Fig. 4a).

Solution and MAS NMR experiments further demonstrate that the spin-X-isolated Aβ oligomers are conformationally diverse and grow over time, yet maintain their disordered nature. The 1D profiles of ^1^H MAS spectra do not change significantly over a period of 16 days, indicating the general fold of the oligomer is preserved. Between 4 and 9 days, the two sharp peaks at 1.20 and 1.18 ppm broaden beyond detection, while at the same time the oligomer peak at 0.16 ppm undergoes similar line-broadening (Supplementary Fig. 7). We attribute this line-broadening to the increasing size of disordered Aβ oligomers. Even after 4 days of aggregation, cross-peaks were not observed in 2D NOESY spectra under MAS conditions (Supplementary Fig. 8).

### RFDR reveals details that solution NMR cannot for intermediate size oligomers

Since the RFDR pulse sequence (Supplementary Fig. 3) utilizes the transfer of proton magnetization via coherent ^1^H-^1^H dipolar couplings and an incoherent cross-relaxation from the NOE to generate cross-peaks^34^, we would expect the RFDR pulse sequence to be more sensitive to larger oligomers than the traditional NOESY experiment utilizing only NOE cross-relaxation. Accordingly, cross-peaks were not observed in 2D NOESY spectra obtained under MAS conditions and very few peaks were observed under static conditions (Supplementary Fig. 8), indicating the dipolar interaction among protons dominates the transfer of magnetization between nuclei of the disordered Aβ_1-40_ oligomer as evidenced by the RFDR spectrum in Fig. 3 (blue).

**Figure 5:**
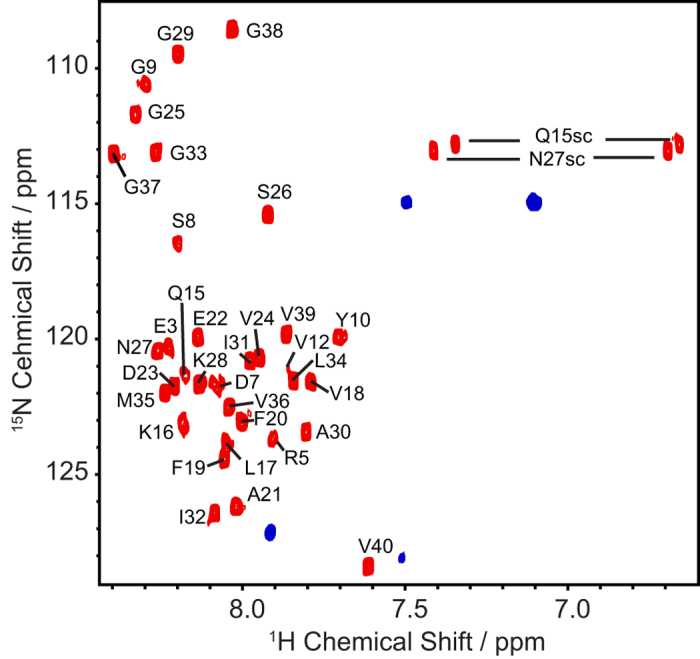
Comparison of ^1^H/^15^N HSQC spectra of the freshly dissolved (red) and the spin-X-isolated disordered oligomer (blue) of Aβ_1-40_ (after 4 days) recorded from a 900 MHz spectrometer. Both experiments were performed in 10 mM phosphate buffer, pH 7.4, and 10% D_2_O at 25 °C.

We further tested this observation by comparing the results from the RFDR experiment to solution NMR experiments using the time dependent growth of the oligomer from 4 to 19 days (Fig. 4c,d). Static TOCSY spectra (Fig. 4c) over this timeframe showed a severe decrease in the number of cross-peaks observed, while NOESY experiments under static conditions yielded little to very few cross-peaks at 4 days (Fig. 4d) and no cross-peaks were observed for the NOESY experiment at 19 days (data not shown). Similarly, 2D ^1^H/^15^N heteronuclear single-quantum correlation (HSQC) experiments performed on the spin-X-isolated oligomer of Aβ_1-40_ after 4 days (Fig. 5) exhibit drastically different HSQC spectra than that observed for a freshly dissolved Aβ_1-40_ sample^41^,^42^. Only four peaks are observed in the HSQC spectrum of the isolated, disordered Aβ_1-40_ oligomer after 4 days of aggregations; these likely coming from highly mobile residues and/or mobile side-chains. These results then suggest that molecular motions do not average out the ^1^H-^1^H dipolar interaction of the oligomer, and therefore the peaks are broadened beyond detection in solution NMR experiments. We therefore conclude that while the oligomer is observable by traditional solution NMR experiments, only limited information can be acquired, in contrast to the high-resolution information obtained from the RFDR-based 2D ^1^H/^1^H MAS solid-state NMR experiment.

## Conclusion

**Figure 6:**
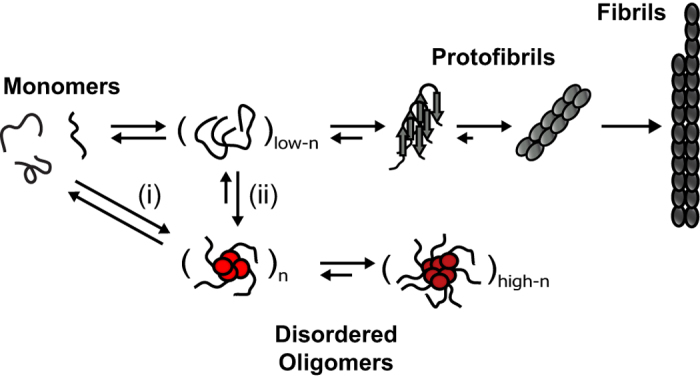
Simultaneously occurring aggregation pathways of Aβ_1-40_. Early aggregates maintain structural similarity to the stable, disordered Aβ_1-40_ oligomers observed at late aggregation stages. The early aggregates either (i) solely nucleate the disordered oligomers or (ii) act as a single nucleating seed from which the two distinct aggregation pathways bifurcate.

Using the ^1^H/^1^H RFDR technique, we were able to reveal the dynamic and disordered structure comprised of turns and twists for the intermediate size (5–10 nm) oligomers. By implementing both solution- and solid-state NMR experiments, particularly through the ^1^H-^1^H dipolar couplings recoupled by RFDR, we have characterized high-resolution structural properties of a dynamic and disordered Aβ_1-40_ oligomer and the development of early amyloid aggregates. Disordered and/or micelle-like structures have been observed for other amyloid-forming proteins and peptides as well^43^,^44^,^45^,^46^. In terms of a stable conformation, the oligomers studied here resemble, at least biophysically, conformers generated by small molecule amyloid inhibitors (such as polyphenols like EGCG^41^,^47^ or resveratrol^48^); i.e., inert Aβ species that are large and predominantly unstructured. However, the stable oligomer studied here occurs without small molecule perturbations or chemical modifications in the Aβ peptide sequence. Furthermore, this disordered oligomer forms simultaneously with the highly ordered and well- structured β-sheet fibrils, indicating that a single aggregation pathway is not necessarily prevalent for a given preparation of Aβ (Fig. 6). Whether the disordered Aβ oligomer studied here is cytotoxic remains to be determined; however, the fact that this disordered conformation persists to an end-state that is not fibrillar is unexpected in light of the concept of nucleated conformational conversion and Aβ_1-40_ aggregation pathways in general^49^,^50^.

In a more general sense, our results demonstrate the value of the RFDR-based 2D ^1^H/^1^H experiment in obtaining high-resolution information on supramolecular assemblies not easily amenable to analysis by other biophysical techniques, including solution NMR and other solid-state NMR experiments. Specifically, most of the high resolution data so far on amyloid oligomers and fibers has come from solid-state NMR experiments. These experiments have been invaluable in advancing our understanding of amyloid and other supramolecular assemblies. However, solid-state NMR experiments are inherently insensitive and require frozen or lyophilized samples that must also be isotopically labeled, often in a site-specific fashion that requires chemical synthesis rather than recombinant expression. Importantly, the ^1^H/^1^H RFDR experiment can be run on aggregated solution samples using proton detection. For this reason, it is more sensitive than most other solid-state NMR experiments but is able to access a size range inaccessible to solution NMR experiments. Since it is run under solution conditions that allow dynamic averaging and very large oligomeric species are spectrally filtered out by the procedure, it is also very tolerant to both conformational heterogeneity and heterogeneous oligomer size distributions. As demonstrated in this study, the signal from the oligomer can be resolved without purification even though the oligomer comprises only 5% of the total sample. Finally, the experiment does not require isotopic labeling. All of these characteristics make it ideal for medically relevant samples that have been difficult to characterize, such as amyloid oligomers directly derived from the brain, which in some cases have shown intriguingly different properties than the corresponding recombinant or synthetic Aβ peptide^13^,^51^,^52^,^53^,^54^.

## Methods

### Peptide Synthesis

Aβ_1-40_ was synthesized manually by solid-phase Fmoc-based chemistry using the dimethoxybenzyl-protected (dmn) dipeptide, Fmoc-Val-(Dmb)-Gly-OH at positions 36 and 37 for the purpose of preventing aggregation during synthesis. The peptide was cleaved from the resin using 92.5% trifluoroacetic acid (TFA), 2.5% H_2_O, 2.5% ethanedithiol, and 2.5% anisole. The crude peptide was dissolved in 20% acetic acid (v/v) and purified by reverse-phase HPLC using a Waters semipreparative C18 column equilibrate in 0.1% TFA. The peptide was eluted with a linear gradient of 0–80% acetonitrile at a flow rate of 10 ml/min. Proper synthesis and purification were validated using matrix-assisted laser desorption ionization mass spectrometry, which gave a value corresponding to the correct mass 4329.9 Da.

### Sample Preparation

To remove preformed aggregates, the purified peptide was dissolved in 1% ammonium hydroxide (v/v) at a concentration of 1 mg/ml followed by removal of the solvent by lyophilization for 24 hours in aliquots of either 0.1 or 0.3 mg. The aliqouted peptide was then stored at −20 °C and only used once.

#### Preparation of Aggregated Aβ_1-40_ Sample

For preparation of Aβ_1-40_ aggregated sample containing a mixture of fibers and disordered oligomers, 0.1 or 0.3 mg of the lyophilized peptide was solubilized in a 10 mM sodium phosphate buffer (pH 7.4) solution at a concentration of 1 mg/ml (231 μM) and incubated for 48 hours at 37 °C under agitation at 1000 rpm. This aggregated Aβ_1-40_ sample was then used to seed 5% of the total concentration of freshly dissolved Aβ_1-40_ in the same buffer conditions at a total Aβ_1-40_ concentration of 1 mg/ml. The seeded Aβ_1-40_ sample was incubated for 48 hours at 37 °C under agitation at 1000 rpm to form a sample containing a minority of Aβ_1-40_ oligomers (17.0 ± 6.0 μM or ~10% of the total peptide concentration) amongst a much larger population of Aβ_1-40_ fibrils. Concentrations were determined by the Thermo-Scientific BCA protein assay kit from 3 independent samples.

#### Isolation of Disordered Oligomers

Upon completion of preparing the seeded Aβ_1-40_ aggregates (i.e. at the end of the 4 day incubation period), disordered oligomers were isolated using a Spin-X microcentrifuge spin column (Corning Inc.), containing a 0.22 μm cellulose acetate filter. The filtrate contained the isolated Aβ_1-40_ disordered oligomer (the 4-day old oligomer) and the Aβ_1-40_ fibrils were retained in the retentate. Due to the concentrations of the disordered oligomers being very low for NMR measurements, these samples could be lyophilized and rehydrated at double their concentration (quantified above). This had no effect on the solution characteristics of the sample, namely its size, morphology, or secondary structure as verified by CD and DLS.

#### Monomer preparation (i.e., Freshly Dissolved Aβ_1-40_)

Preparation of Aβ_1-40_ monomer sample was performed as described previously^14^. Briefly, 0.1 mg of the lyophilized peptide was first dissolved in 10 μl of 1 mM NaOH and sonicated until the peptide was solubilized. The peptide solution was then hydrated in H_2_O (or 100% D_2_O for MAS NMR measurements), buffered in 10 mM sodium phosphate (pH 7.4) and diluted to 76 μM (0.1 mg in 300 μl).

### NMR Spectroscopy

All NMR data was processed using TopSpin 2.1 (Bruker). 1D data were analyzed using TopSpin 2.1 and 2D data were analyzed using SPARKY. In all NMR spectra, the ^1^H peak from H_2_O was used as a chemical shift reference by setting its frequency at 4.7 ppm.

#### MAS NMR Spectroscopy

MAS NMR experiments were performed at 298 K or 310 K on an Agilent/Varian VNMRS 600 MHz solid-state NMR spectrometer using a 4 mm ^1^H/X double-resonance Nanoprobe. The spectrometer was operated with a deuterium field lock and a MAS spinning speed of 2.7 kHz. The proton carrier frequency was set to the resonance frequency of water for all experiments and ^1^H_2_O signal was suppressed using a 10 Hz saturation RF pulse for 1 s at the beginning of NOESY or RFDR-based 2D ^1^H/^1^H experiments. The radio frequency field strength used for the 90° and 180° pulses was 61 kHz. The NOESY and RFDR-based 2D ^1^H/^1^H spectra were recorded using 1100 scans, 200 t_1_ increments, 6252 t_2_ complex points and a spectral width of 11 ppm in both frequency dimensions. The experimental data sets were zero-filled in both t_1_ and t_2_ dimensions to form a 2048 × 4096 data matrix. Phase shifted sine bell multiplication was applied to both dimensions prior to Fourier transformation. Since the maximum MAS rate possible with the Agilent Nanprobe is 2.7 kHz, to achieve higher MAS rates while maintaining the utility of deuterium locking and pulse-field gradients, we used a Bruker complete multi-phase (CMP) probe on a Bruker 600 MHz Avance III NMR spectrometer. 1D ^1^H NMR spectra were acquired for MAS speeds from 5 to 15 kHz (Supplementary Figure 5). RFDR-based 2D ^1^H/^1^H experiments were performed under 10 kHz MAS with mixings times of 20 and 50 ms and using non-uniformly sampled (NUS), interleaved datasets. NUS datasets, where each data is split into its 2 component dataets, was done using the Split program and processed separately with the standard ‘xfb’ command, regenerating the missing points and transforms the datasets.

Assignment of proton resonances was done using SPARKY with published assignments for Aβ_1-40_ as a guide. 1D ^1^H MAS experiments were recorded with 20000 scans, 16 dummy scans, a spectral width of 12 ppm, and an acquisition time of 0.5 s. The proton carrier frequency was set at water resonance for all experiments and ^1^H_2_O resonance was suppressed using a 50 Hz RF pulse for 1.5 s.

#### Solution NMR Spectroscopy

Solution NMR spectroscopy was performed on the filtered oligomer. Data were acquired on a 900 MHz Bruker NMR Spectrometer equipped with a cryogenic triple-resonance pulse-field gradient probe. 1D and 2D NMR spectra were collected at either 298 K or 310 K. NOESY spectra were acquired with a spectral width of 12 ppm in both dimensions, with 400 (ω_1_) and 2048 (ω_2_) complex points using a 1.5 s recycle delay. The NOESY experiments were acquired with two different mixing times: 250 and 600 ms. Solvent suppression was done using gradient pulses centered at ^1^H resonance frequency of water. The same parameters were used for TOCSY experiments; however, mixing times of 70 and 100 ms were used. The experimental data sets were zero-filled to form a 2048 × 4096 data matrix and a phase-shifted sine bell multiplication was applied to both dimensions prior to Fourier transformation. ^15^N-labeled Aβ_1-40_ was purchased from rPeptide (Athens, GA, U.S.A.) and used for HSQC experiments. The exact same sample preparation protocols detailed above were used for ^15^N-labeled Aβ_1-40_ samples. Each spectrum was obtained from 128 t1 experiments, 92 scans (for the spin-X-isolated Aβ_1-40_ oligomer) and 1 s recycle delay.

### Circular Dichroism (CD)

CD measurements were performed on JASCO J-715 Spectropolarimeter using a 0.1 cm path length cell. Isolated oligomer and fibril samples of Aβ_1-40_ were prepared as described above. Molar CD per residue values were calculated using *ε* = *θ*_*obsd*_/(3298*lcn*), where *θ*_*obsd*_ is the observed ellipticity measured in millidegrees, *c* is the molar concentration, *l* is the cell path length in centimeters, and *n* is the number of residues in the peptide.

### bis-ANS and Thioflavin T Fluorescence Assays

Aβ_1-40_ fibril formation was measured by increased fluorescence emission upon binding of amyloid fibers to the commonly used amyloid-specific dye, thioflavin T (ThT). Aβ_1-40_ oligomer and fibril formation were also measured by fluorescence emission spectra of the less specific dye, 4,4′-Dianilino-1,1′-Binaphthyl-5,5′-Disulfonic Acid (bis-ANS); purchased from Santa Cruz Biotechnology, Inc. bis-ANS exhibits limited fluorescence in water; however, becomes considerably fluorescent upon binding hydrophobic surfaces. Isolated oligomeric and fibrillar Aβ_1-40_ species were prepared as described above. The retentate containing Aβ_1-40_ fibrils was dissolved in 200 μL of 10 mM sodium phosphate buffer, pH 7.4, of which 90 μL was then aliquoted into microcentrifuge tubes. Similarly, the filtrate containing the Aβ_1-40_ oligomers was aliquoted into 90 μL quantities as well. Either ThT or bis-ANS fluorescent dye was then added to a peptide aliquot at a concentration of 10 μM and fluorescence emission was measured on a Horiba FluoroMax 4 spectrofluorometer. An excitation wavelength of 446 nm and 350 nm was used for ThT and bis-ANS, respectively.

### Atomic Force Microscopy (AFM)

An aggregated Aβ_1-40_ sample prepared as described above was deposited onto freshly cleaved mica and incubated for twenty minutes at room temperature. The surfaces was rinsed with nanopure water and dried under nitrogen flow. Dry imaging was carried out in tapping mode using a Nanoscope III atomic force microscopy (AFM) and JZ Scanner (Veeco) with VistaProbes T300R (NanoScience, AZ; nominal radius 10 nm, force constant 40 N/m, resonance frequency 300 kHz). The AFM was calibrated with a 100 nm x 100 nm standard (2D-100, NANOSENSORS, Switzerland). After calibration, the percent error was −0.6%. Random locations on the sample were selected for imaging. Particles were detected and height measurements were made using SPIP 6.0.13 software (NanoScience Instruments).

### Dynamic Light Scattering (DLS)

Light scattering experiments were performed on Aβ_1-40_ samples prepared as described using a DynaPro Nanostar instrument from Wyatt Technology (Santa Barbara, CA). Light scattering was measured at 90°. The intensity correlation function and the distribution of the hydrodynamic radii (R_H_) of the particles contributing to the scattering were determined using DYNAMICS software (Wyatt Technology).

### Transmission Electron Microscopy (TEM)

Samples for negative stain TEM analysis were deposited on continuous carbon films on copper rhodium 100 mesh grids (Electron Microscopy Sciences, EMS Hatfield PA.). Prior to adding samples, the grids were charged using a glow discharger for 15 s at 30 mA negative discharge. Fibrillar and oligomer sample solutions at 1 mg/ml were adsorbed to the grids for 2 minutes prior to rinsing with two 10 μL drops of water for 10 s. Samples were blotted using No. 2 Whatman filter paper. Samples for TEM were then stained with a 10 μL drop of freshly filtered 2% uranyl acetate (EMS) for 15 s before blotting excess stain. Samples were analysed using a Philips CM-100 microscope operating at 80 kV.

## Additional Information

**How to cite this article**: Kotler, S. A. *et al.* High-resolution NMR characterization of low abundance oligomers of amyloid-ß without purification. *Sci. Rep.* **5**, 11811; doi: 10.1038/srep11811 (2015).

## Supplementary Material

Supplementary Information
