# Supplementary material for: High-resolution NMR characterization of low abundance oligomers of amyloid-β without purification
Source: Sci Rep. 2015 Jul 3;5:11811. doi: 10.1038/srep11811 (PMC4490348; doi:10.1038/srep11811)
Supplement: Supplementary Information [file srep11811-s1.pdf]

Supplementary Figures

High-resolution NMR characterization of low abundance oligomers of amyloid-β without purification

Samuel A. Kotler[1], Jeffrey R. Brender[1,2], Subramanian Vivekanandan[1], Yuta Suzuki[2], Kazutoshi Yamamoto[1,2], Martine Monette[4], Janarthanan Krishnamoorthy[1,2], Patrick Walsh[1,2], Meagan Cauble[2], Mark M. Banaszak Holl[2,3], E. Neil. G. Marsh[2], and Ayyalusamy Ramamoorthy[1,2*]

[1]Biophysics, [2]Department of Chemistry, and [3]Michigan Nanotechnology Institute for Medicine and Biological Sciences, University of Michigan-Ann Arbor, Ann Arbor, Michigan 48109, U.S.A.

[4]Bruker BioSpin Ltd., Bruker Corporation, 555 E Steeles Ave, Milton, ON, Canada

* Corresponding Author:

Ayyalusamy Ramamoorthy

930 N. University Avenue, Ann Arbor, Michigan 48109-1055 (USA)

Phone: +1 734 647-6572; Fax: +1 734 615-3790 E-mail: ramamoor@umich.edu

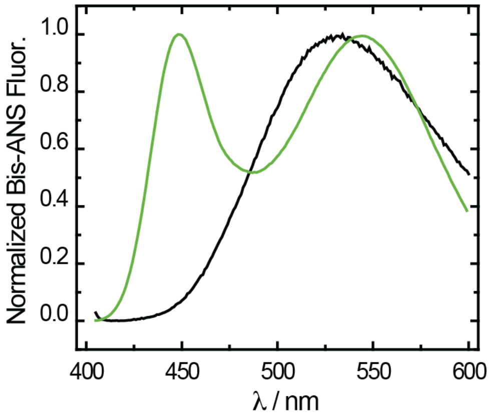

Supplementary Figure 1. Characterizing the aggregated Aβ_{1-40} sample. The bis-ANS fluorescence assay was used to compare normalized bis-ANS fluorescence in solution (black) to its emission spectrum upon binding isolated Aβ_{1-40} fibrils (green).

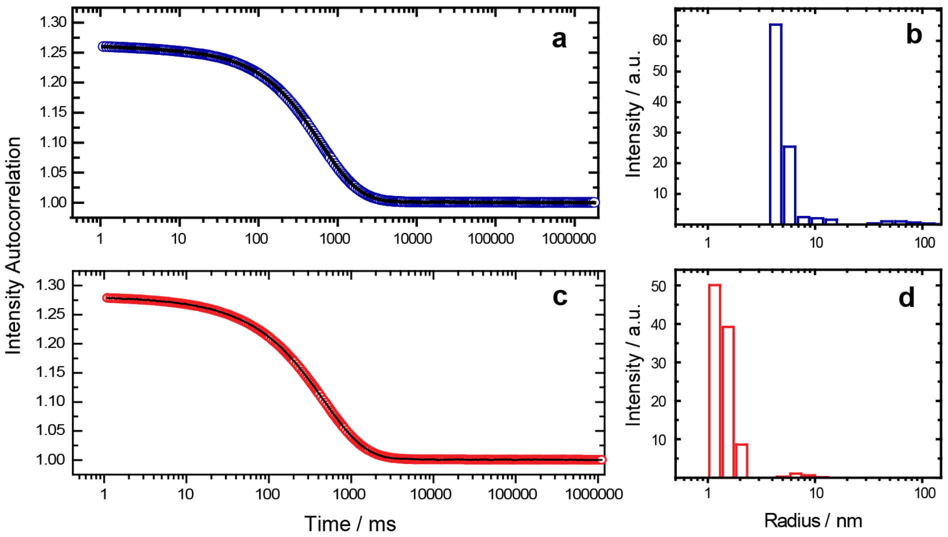

Supplementary Figure 2. DLS studies on the spin-X-isolated oligomer and freshly dissolved samples of A$\beta_{1\text{-}40}$. (a, b) The autocorrelation (a, blue circles) with a regularization fit and the corresponding distribution (b) of hydronamic radii obtained from the fitted autocorrelation of the spin-X isolated oligomer. DLS studies on the freshly dissolved A$\beta_{1\text{-}40}$ sample (c, d) showing that the initial population of A$\beta_{1\text{-}40}$ is primarily monomeric with some low order oligomers (dimers or trimers). This is apparent through a regularization fit of the autocorrelation (c) yielding a distribution of hydronamic radii (d) of the freshly dissolved A$\beta_{1\text{-}40}$ sample.

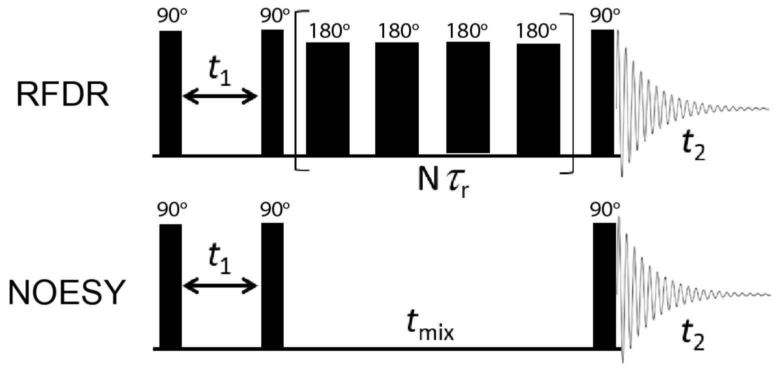

Supplementary Figure 3. The two-dimensional RFDR-based (top) and NOESY (bottom) $^1H/^1H$ chemical shift correlation experiments used in this study.

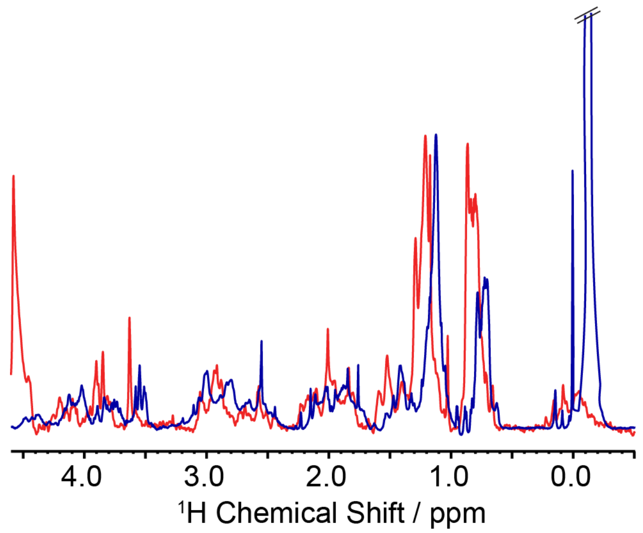

Supplementary Figure 4. The aggregated A$\beta_{1\text{-}40}$ sample shows strong up-field shifts. An overlay of the 1D ^{1}H MAS NMR spectra of freshly dissolved A$\beta_{1\text{-}40}$ (red) and the unfiltered, disordered A$\beta_{1\text{-}40}$ oligomer (blue) acquired at 37 °C under 2.7 kHz MAS in 100% D_2O, 10 mM sodium phosphate buffer, pH 7.4.

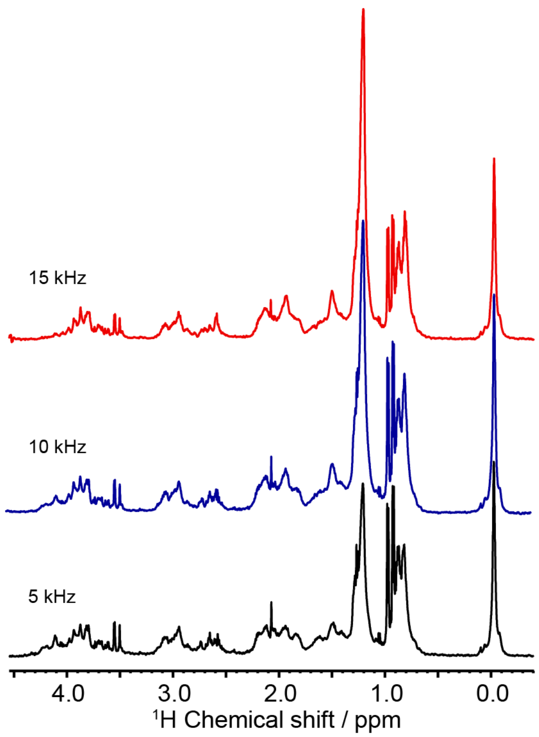

Supplementary Figure 5. 1D ^{1}H MAS spectra acquired under increasing MAS rates on the filtered disordered $A\beta_{1\text{-}40}$ oligomer. Spectra were acquired at 600 MHz in 100% D_2O, 10 mM sodium phosphate buffer, pH 7.4, at 25 °C. The filtered oligomer sample was lyophilized and re-hydrated to double its initial concentration, making for a total $A\beta_{1\text{-}40}$ of ~35 μM.

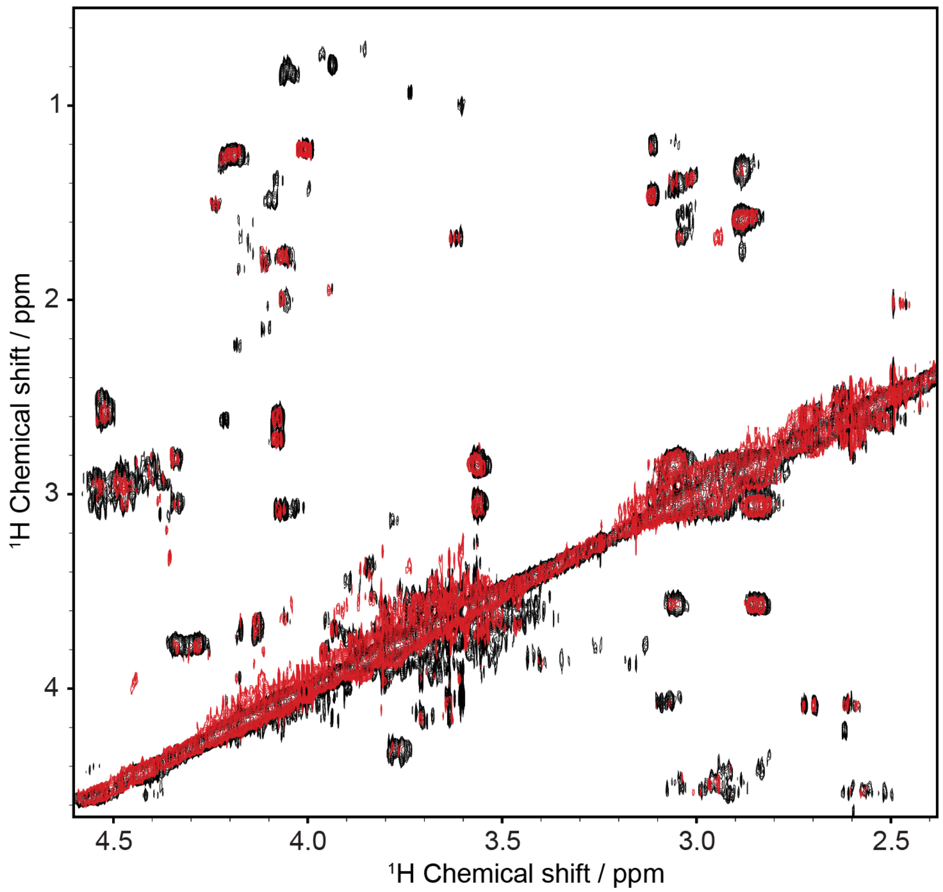

Supplementary Figure 6. An overlay of the aliphatic and alpha proton regions of RFDR-based 2D $^1H/^1H$ spectra acquired using mixing times of 20 ms (red) and 50 ms (black) on the aggregated $A\beta_{1-40}$ sample. The spectra were acquired at 37 °C with an MAS rate of 2.7 kHz in 100% D_2O, 10 mM phosphate buffer, pH 7.4. The slow MAS rate used here serves to insufficiently average the dipole-dipole interactions of the disordered oligomer such that the RFDR pulse sequence can be used to recouple its "residual" dipolar couplings under MAS conditions. The fact that a well-resolved RFDR-based 2D $^1H/^1H$ chemical shift correlation spectrum is obtained strongly suggests that this soluble, oligomer species is sufficiently large for the usage of such recoupling methods. Additionally, the low MAS rates insufficiently averages the ^1H-^1H dipolar couplings of the fibrillar species present in the aggregated $A\beta_{1-40}$ sample. Thus, the RFDR-based 2D $^1H/^1H$ methodology effectively acts as a filter for intermediate aggregates of $A\beta_{1-40}$. These spectra were assigned by using the 2D $^1H/^1H$ TOCSY spectrum of the filtered oligomer under both static and MAS conditions (see figures S4 and S7). Assignments of the two TOCSY spectra were guided by a previously assigned spectrum in the following study: Vivekanandan et al. BBRC, 2011, 411, 312-16.

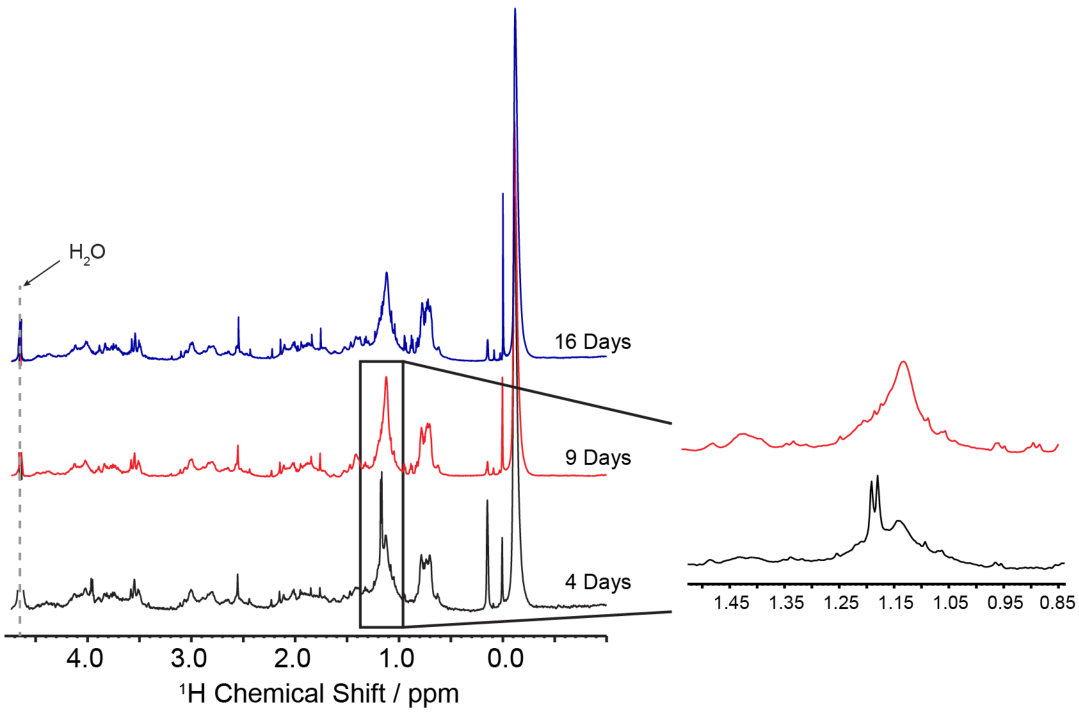

Supplementary Figure 7. Time-course 1D ^{1}H MAS NMR spectra of the aggregated A$\beta_{1\text{-}40}$ sample, containing the disordered oligomer. Little difference is observed in the 1D ^{1}H spectral profiles over the same timeframe for which the RFDR-based 2D ^{1}H/^{1}H experiments were performed, indicating the disordered oligomer is stable over this time period. The initial time point of 4 days marks the completion of sample preparation period described in the experimental procedures and the starting point for all subsequent measurements performed on the disordered A$\beta_{1\text{-}40}$ oligomer. While few changes are seen over this 16-day period, between 4 days and 9 days there are several distinct changes occurring. The oligomer peaks ca. 0.15 ppm undergoes a drastic loss of signal between 4 and 9 days. Additionally, the doublet ca. 1.2 ppm is broadened beyond detection. Collectively, this would suggest 1) a heterogeneous mixture of A$\beta_{1\text{-}40}$ oligomer species present at 4 days and 2) that the disordered oligomer(s) is growing in size over the course of 16 days.

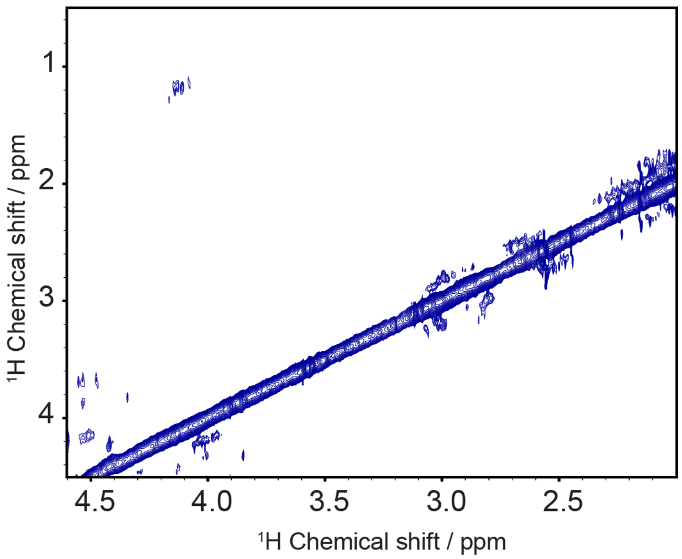

Supplementary Figure 8. Two-dimensional $^1H/^1H$ NOESY experiments under 2.7 kHz MAS on the mixed fiber and oligomer sample of A$\beta_{1\text{-}40}$. The spectrum was acquired with a 50 ms mixing time at 600 MHz in 100% D_2O, 10 mM sodium phosphate buffer, pH 7.4, at 37 °C, and corresponds to the same sample used in Fig. 2 of the main text.
